# Supplementary material for: Allosteric modulation of LRRC8 channels by targeting their cytoplasmic domains
Source: Nat Commun. 2021 Sep 14;12:5435. doi: 10.1038/s41467-021-25742-w (PMC8440666; doi:10.1038/s41467-021-25742-w)
Supplement: Supplementary file 1 — Supplementary Information [file 41467_2021_25742_MOESM1_ESM.pdf]

Supplementary Information

Allosteric modulation of LRRC8 channels by targeting their cytoplasmic domains

Dawid Deneka, Sonja Rutz, Cedric A. J. Hutter, Markus A. Seeger, Marta Sawicka and Raimund Dutzler

Supplementary Figures

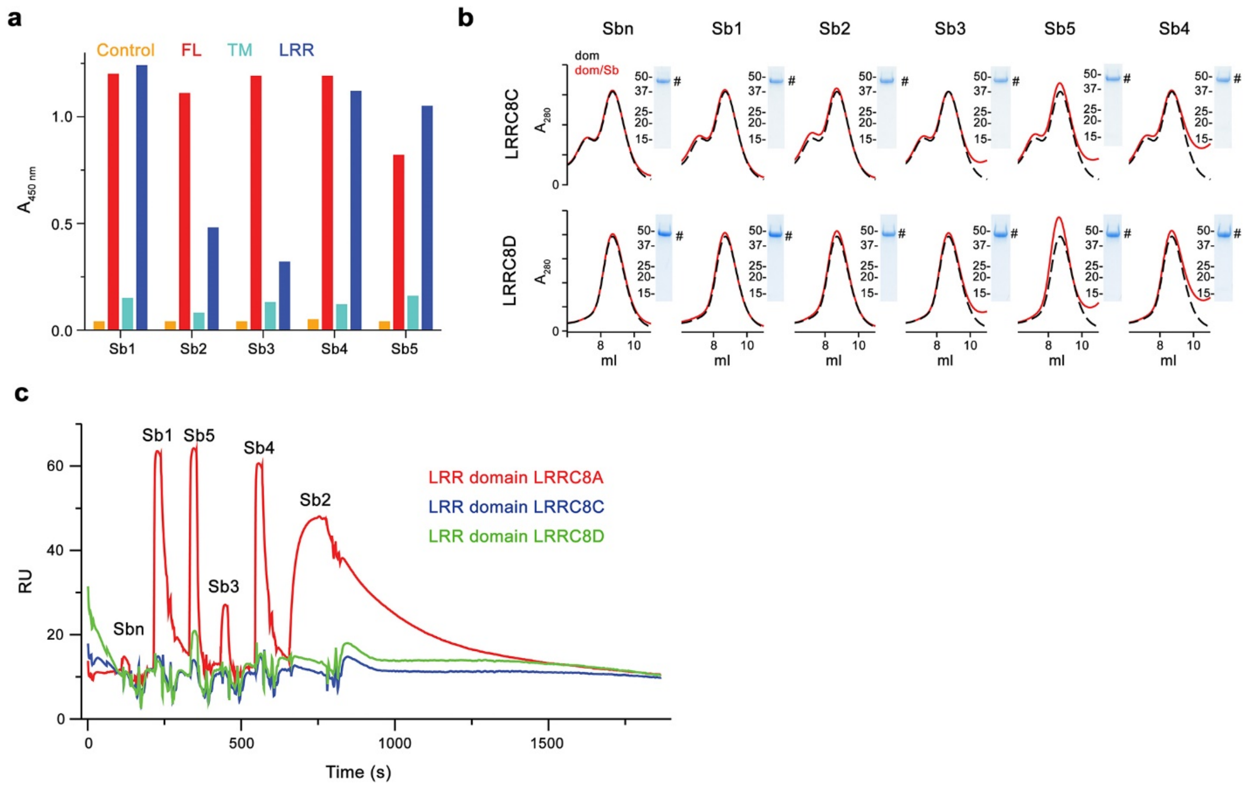

Supplementary Fig. 1: Selection and characterization of sybodies targeting LRRC8A. a ELISA-derived signals of respective sybodies against different parts of the LRRC8A channel. Control refers to wells coated with the control protein AcrB, FL to the full-length LRRC8A channel, TM to its isolated transmembrane domain and LRR to its isolated LRR domain. **b** Sections of size-exclusion chromatograms showing the elution of the LRR domains of LRRC8C (top) and LRRC8D (bottom, black dashed lines) and of mixtures with respective sybodies (red). Sbn refers to the mixture of the LRRC8A domain with a sybody targeting a protein unrelated to the LRRC8 family. Insets show SDS-PAGE gels of the peak fractions with the LRR domain of LRRC8A indicated (#). No gel showed any band of a co-eluted sybody. Numbers refer to the molecular weight (kDa). **c** Response of the application of different sybodies at a concentration of 1 µM to the immobilized LRR domains of LRRC8A (red), LRRC8C (blue) and LRRC8D (green). The strong signals in the traces with the LRR domain of LRRC8A and the absence of any detectable signal for the corresponding domains of LRRC8C and LRRC8D underlines the specificity of binding.

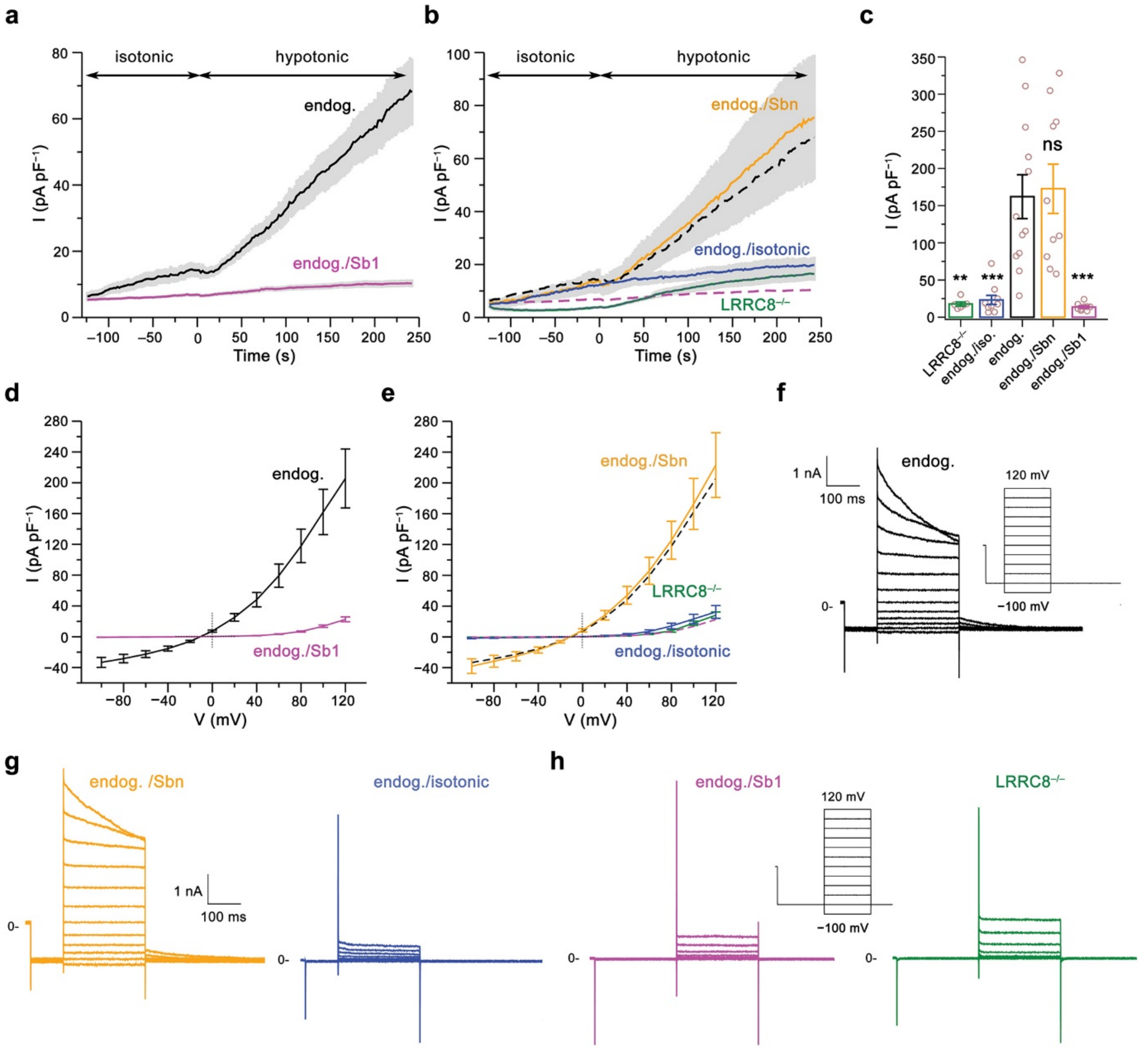

Supplementary Fig. 2: Swelling-activated currents. Average current density of HEK293 cells evoked in response to cell swelling. **a** Currents recorded from non-transfected cells (black) and cells that were transfected with a construct expressing the sybody Sb1 (magenta). **b** Currents from LRRC8$^{-/-}$ cells (green) and cells transfected with the control sybody Sbn (orange). 'Istonic' refers to perfusion with isotonic medium, 'hypotonic' to the switch to hypotonic conditions (t=0). The current response of non-transfected cells under continuous exposure to isotonic conditions is shown for comparison (blue). **c** Current density of individual cells used for the calculation of averages displayed in **a, b**. Values of individual measurements at 100 mV are displayed as circles. Mean current levels are shown as bar. Asterisks indicate significant deviations from WT (endog.) in a two-sided one sample t-test, ns refers to non-significant differences. (LRRC8$^{-/-}$ p=0.0029,

endog./iso.p=0.0004, endog./Sbn p=0.8187, endog./Sb1 p=0.0003). **d** Current–voltage relationships of endogenous swelling-ativated VRAC currents of HEK293 cells. The currents were recorded 250 seconds after a shift to hypotonic conditions (see **a**). **a–e** Averages of independent biological replicates are shown (endog., n=12; endog./Sb1, n = 9; endog./Sbn, n=10; LRRC8$^{-/-}$, n=6; endog./isotonic, n=11), errors are s.e.m. **b, e** Averaged traces displayed in **a** and **d** are shown as dashed lines for reference. **f–h** Representative current traces of activated channels used for plotting the current-voltage relationships displayed in (**d, e**). **f, h** The voltage protocols are shown as inset.

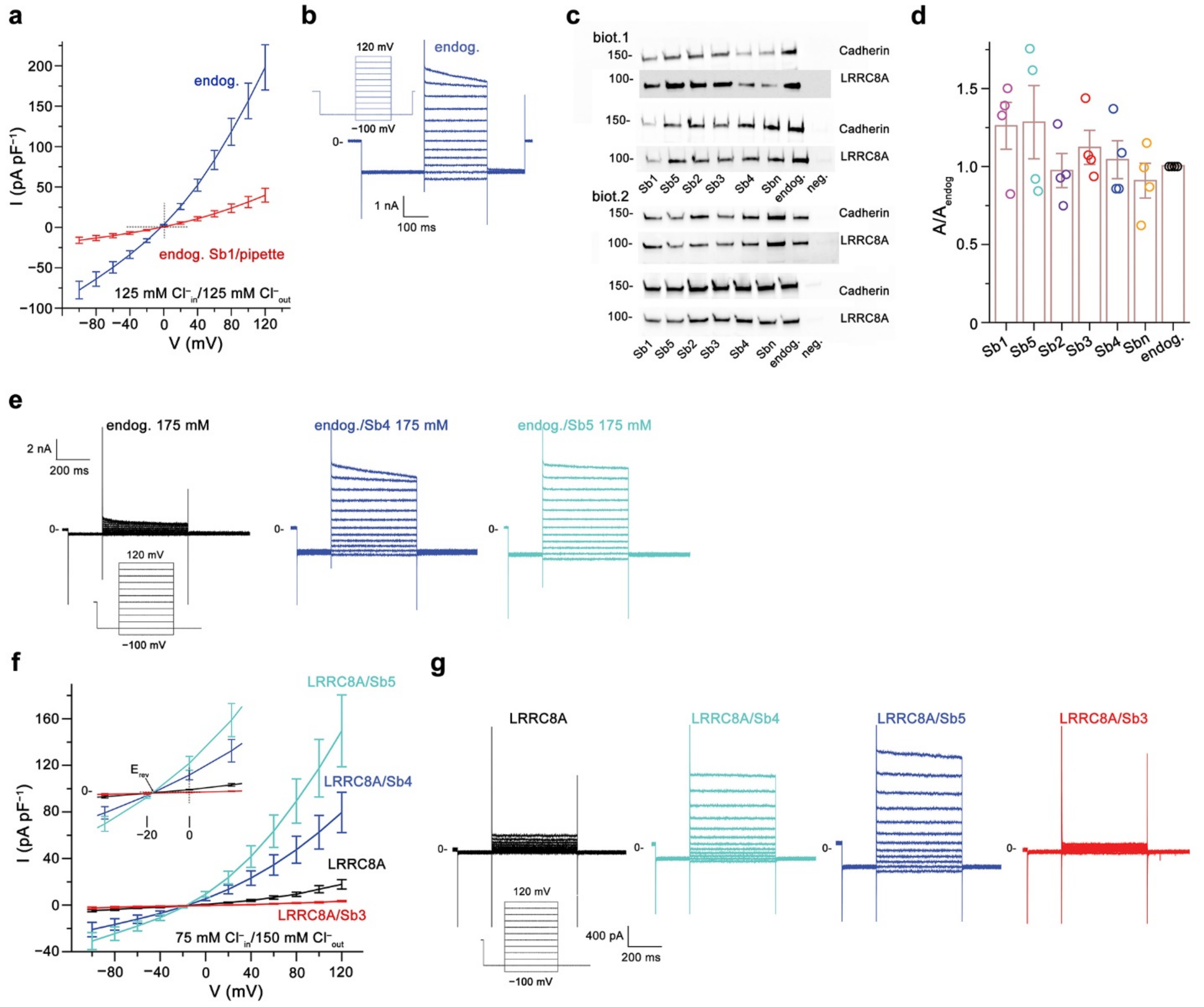

Supplementary Fig. 3: Currents at altered ionic strength and surface expression. a Current–voltage relationships of endogenous VRAC currents of HEK293 cells at symmetric 125 mM Cl^- concentrations. The currents were activated in response to a decreased (125 mM) intracellular monovalent ion concentration and recorded close to steady state 300 seconds after break-in and establishment of the whole-cell configuration (see Fig. 2a). 'endog. Sb1/pipette' refers to endogenous currents recorded with pipette solutions containing 1 µM of the sybody Sb1, which diffuses into the cell to reach its target. Averages of independent biological replicates are shown (endogenous, n = 11; Sb1/pipette, n = 10), errors are s.e.m. **b** Representative current response of activated channels used for plotting the current-voltage relationships displayed in **a**. The voltage protocol displayed as inset (top). **c** VRAC surface expression. Plasma membrane fraction of endogenous LRRC8 channels in HEK293 cells expressing different sybodies obtained from surface biotinylation experiments. Western blots were developed with an antibody targeting

LRRC8A (top) and another antibody targeting pan-cadherin as loading control (bottom). Two independent blots are shown for each of two biological replicates (biot1, biot2). **d** Integrated intensities of specific LRRC8A bands obtained from Western blots displayed in **c**. Data were corrected for the intensity of cadherin quantified on the same blot and normalized to samples of non-transfected cells (endog.). Bars show mean of four blots prepared from two biological replicates of the surface biotinylation experiment. Values from individual experiments are depicted as circles. **e** Representative current response of activated channels used for the generation of the curve shown in Fig. 2e. The voltage protocol is displayed as inset (left). **f** Current–voltage relationships measured from LRRC8$^{-/-}$ expressing LRRC8A and respective sybodies at asymmetric Cl^- concentrations (Cl^-_{in} 75 mM; Cl^-_{out} 150 mM). I–V relationships were recorded 150 seconds after break-in and establishment of the whole-cell configuration. Inset (top left) shows a blowup into the region where currents reverse. E_{rev} is at the Nernst potential of Cl^-. Averages of independent biological replicates are shown (LRRC8A, n = 11; LRRC8A/Sb4, n = 11; LRRC8A/Sb5, n = 12; LRRC8A/Sb3, n = 11), errors are s.e.m. **g** Representative current response of activated LRRC8A channels used for the generation of the curve shown in **f**. The voltage protocol is displayed as inset (left).

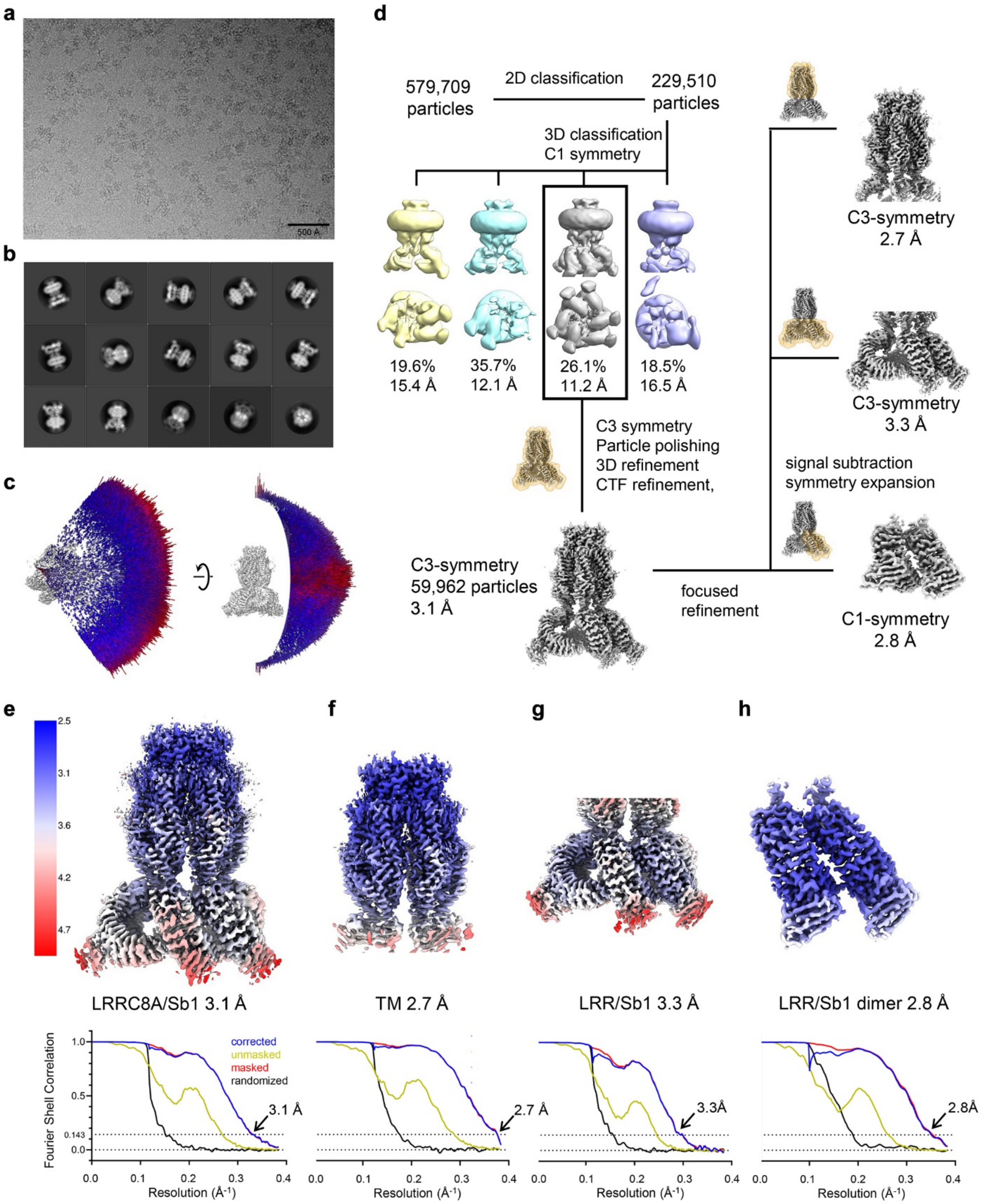

Supplementary Fig. 4: Cryo-EM structure of the LRRC8A/Sb1 complex. **a** Representative cryo-EM micrograph (of a total of 5,494) of the LRRC8A/Sb1 complex dataset. **b** 2D class averages of the LRRC8A/Sb1 complex. **c** Angular distribution plot of all particles included in the

final C3-symmetrized reconstruction. The length and the color of cylinders correspond to the number of particles with respective Euler angles. **d** Data-processing workflow. Four classes generated during 3D classification reveal a well-defined pore domain and structural heterogeneity in the LRR domains, which reflects their intrinsic mobility. Particles assigned to the boxed class with symmetric LRR domains were used in further refinement. The distribution of particles (%) and the resolution of each class is indicated. Application of C3 symmetry and further refinement steps resulted in a reconstruction with an overall resolution of 3.1 Å. The focused refinement of the pore domain improved its resolution to 2.7 Å. Although the extracellular and transmembrane parts of the pore obey a C6 relationship, the symmetry reduces at the intracellular subdomain due to small conformational rearrangements in the regions proximal to the LRR domains and thus C3 symmetry was applied during the refinement. The focused refinement of the LRR domains applying C3 symmetry improved the local resolution of the cytoplasmic components of the channel and yielded cryo-EM density of this region with its bound sybodies Sb1 at 3.3 Å. Symmetry expansion and signal subtraction followed by the focused refinement of a single LRR domain-pair sybody complex with C1-symmetry improved its density further to 2.8 Å, which defined the detailed mode of LRR-LRR domain and LRR-domain sybody interactions. Insets at different steps show the masked regions during refinement. **e**–**h** Final 3D reconstruction colored according to local resolution (top) and FSC plot of the final refined unmasked (yellow), masked (red), phase-randomized (black) and corrected for mask convolution effects (blue) cryo-EM density map of the LRRC8A/Sb1 complex. The resolution at which the FSC curve drops below the 0.143 threshold is indicated. **e** Full-length complex defining the location of sybodies binding to each of the six LRR domains, **f** masked TM region, **g** masked region encompassing all LRR-domains and bound Sb1 sybodies, **h** masked region of a single pair of tightly interacting LRR-domains with bound Sb1 sybodies.

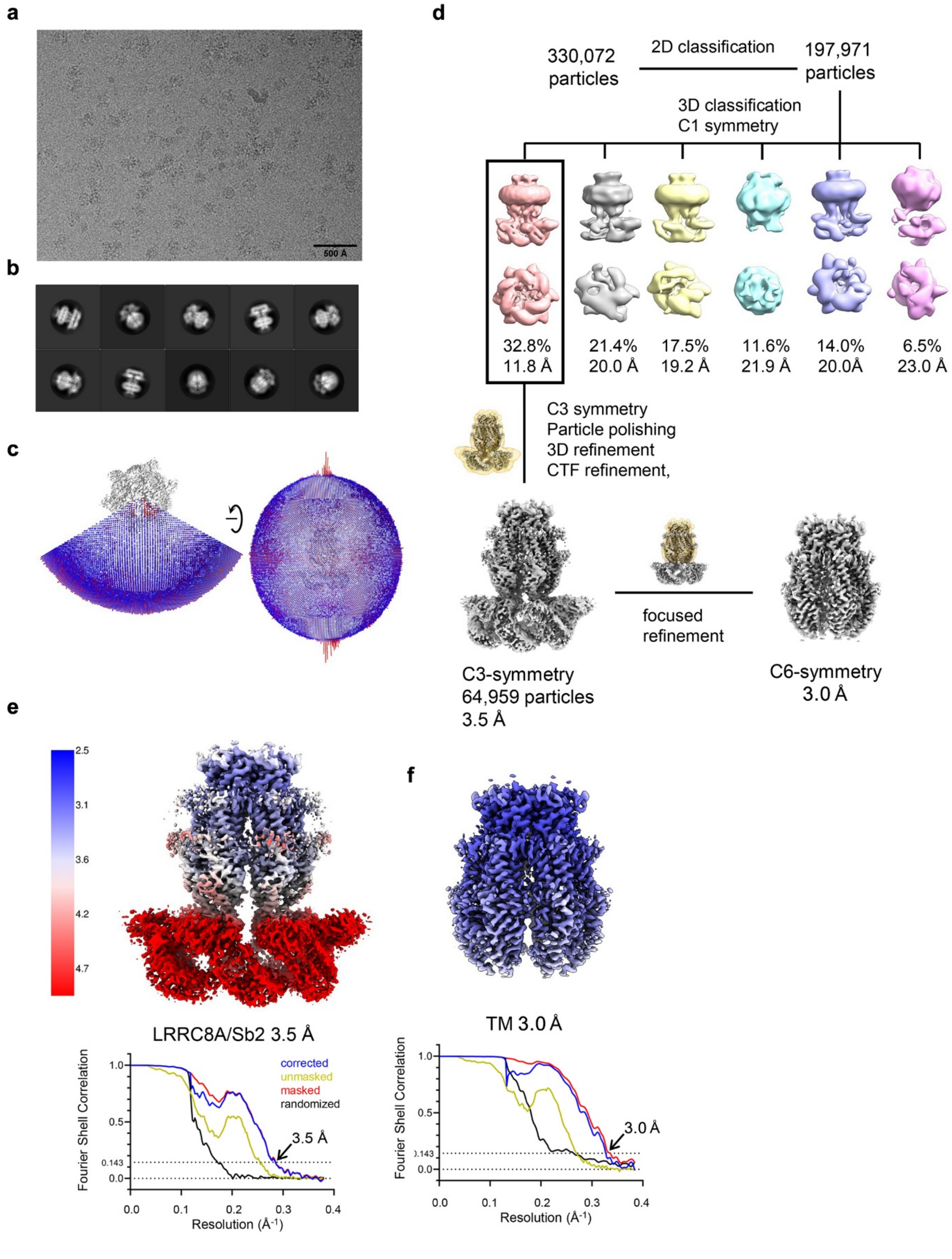

Supplementary Fig. 5: Cryo-EM structure of the LRRC8A/Sb2 complex. **a** Representative cryo-EM micrograph (of a total of 5,633) of the LRRC8A/Sb2 complex dataset. **b** 2D class

averages of the LRRC8A/Sb2 complex. **c** Angular distribution plot of all particles included in the final C3-symmetrized reconstruction. The length and the color of cylinders correspond to the number of particles with respective Euler angles. **d** Data-processing workflow. Six classes generated during 3D classification reveal a well-defined pore domain and pronounced structural heterogeneity in the LRR domains, which reflects their intrinsic mobility. Particles assigned to the boxed class with symmetric LRR domains were used in further refinement. The distribution of particles (%) and the resolution of each class is indicated. Application of C3 symmetry and further refinement steps resulted in a channel with weakly defined LRR domains at an overall resolution of 3.5 Å. The focused refinement of the C6 symmetric pore domain improved its resolution to 3.0 Å. Focused refinement steps of the LRR domains applying C3-symmetry and of a single domain pair with C1-symmetry did not improve the local resolution of the cryo-EM density of cytoplasmic components beyond the density observed in full-length channels and were thus omitted. Insets at different steps show the masked regions during refinement. **e**–**f** Final 3D reconstruction colored according to local resolution (top) and FSC plot of the final refined unmasked (yellow), masked (red), phase-randomized (black) and corrected for mask convolution effects (blue) cryo-EM density map of the LRRC8A/Sb2 complex. The resolution at which the FSC curve drops below the 0.143 threshold is indicated. **e** full-length complex defining the location of sybodies binding to each of the six LRR domains, **f** masked TM region.

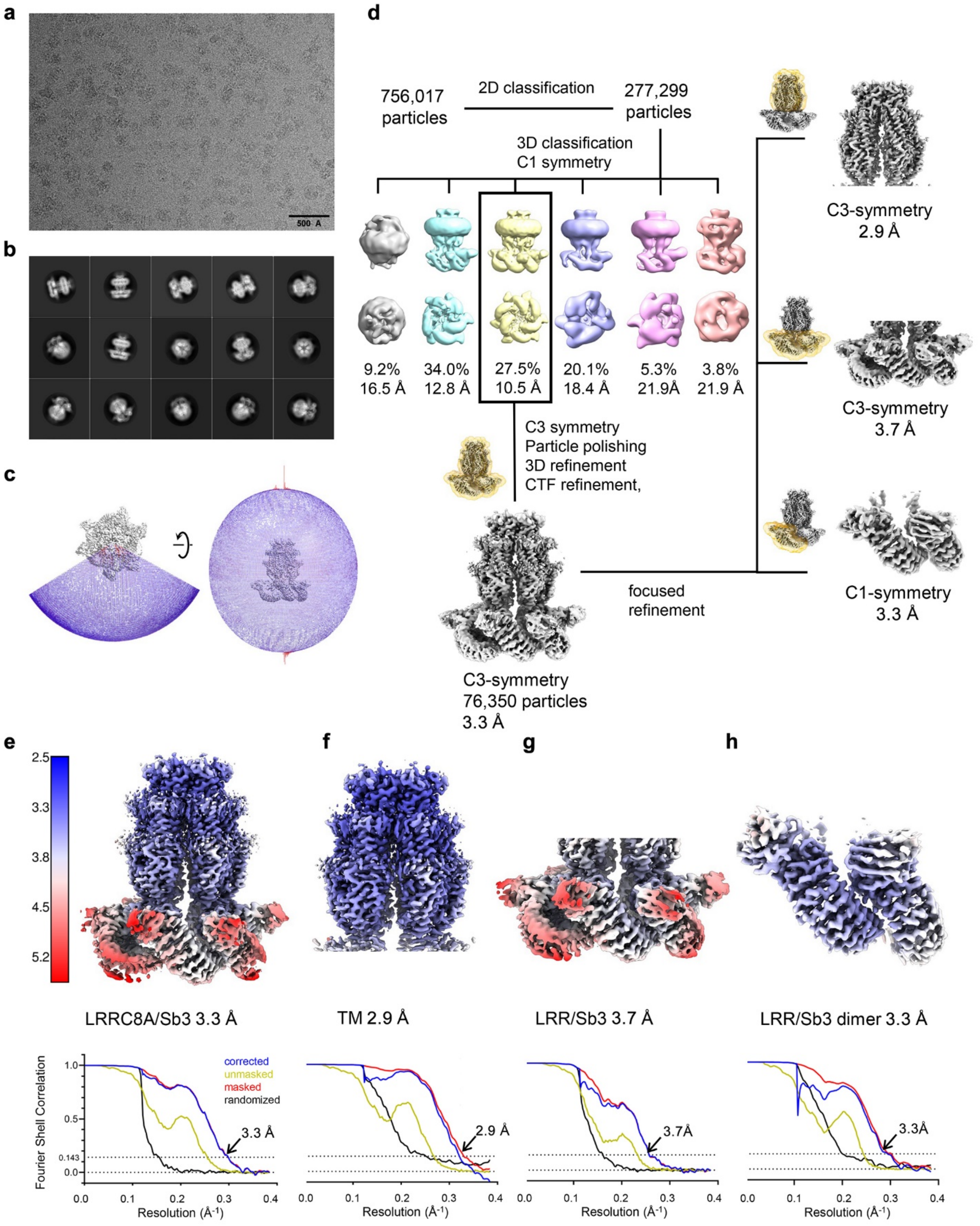

Supplementary Fig. 6: Cryo-EM structure of the LRRC8A/Sb3 complex. a Representative cryo-EM micrograph (of a total of 6,475) of the LRRC8A/Sb3 complex dataset. **b** 2D class averages of the LRRC8A/Sb3 complex. **c** Angular distribution plot of all particles included in the

final C3-symmetrized reconstruction. The length and the color of cylinders correspond to the number of particles with respective Euler angles. **d** Data-processing workflow. Six classes generated during 3D classification reveal a well-defined pore domain and structural heterogeneity in the LRR domains, which reflects their intrinsic mobility. Particles assigned to the boxed class with symmetric LRR domains were used in further refinement. The distribution of particles (%) and the resolution of each class is indicated. Application of C3 symmetry and further refinement steps resulted in a channel with an overall resolution of 3.3 Å. The focused refinement of the C3 symmetric pore domain improved its resolution to 2.9 Å. The focused refinement of the LRR domains applying C3-symmetry improved the local resolution of the cytoplasmic components of the channel and yielded cryo-EM density of this region including the bound sybodies Sb3 at 3.9 Å. Symmetry expansion and signal subtraction followed by the focused refinement of a single LRR domain-pair sybody complex with C1-symmetry improved its density further to 3.3 Å, which defined the detailed mode of LRR-LRR domain and LRR-domain sybody interactions. Insets at different steps show the masked regions during refinement. The particle signal outside the mask was subtracted. **e**–**h** Final 3D reconstruction colored according to local resolution (top) and FSC plot of the final refined unmasked (yellow), masked (red), phase-randomized (black) and corrected for mask convolution effects (blue) cryo-EM density map of the LRRC8A/Sb3 complex. The resolution at which the FSC curve drops below the 0.143 threshold is indicated. **e** full-length complex defining the location of sybodies binding to each of the six LRR domains, **f** masked TM region, **g** masked region encompassing all LRR-domains and bound Sb3 sybodies, **h** masked region of a pair of tightly interacting LRR-domains with bound Sb3 sybodies.

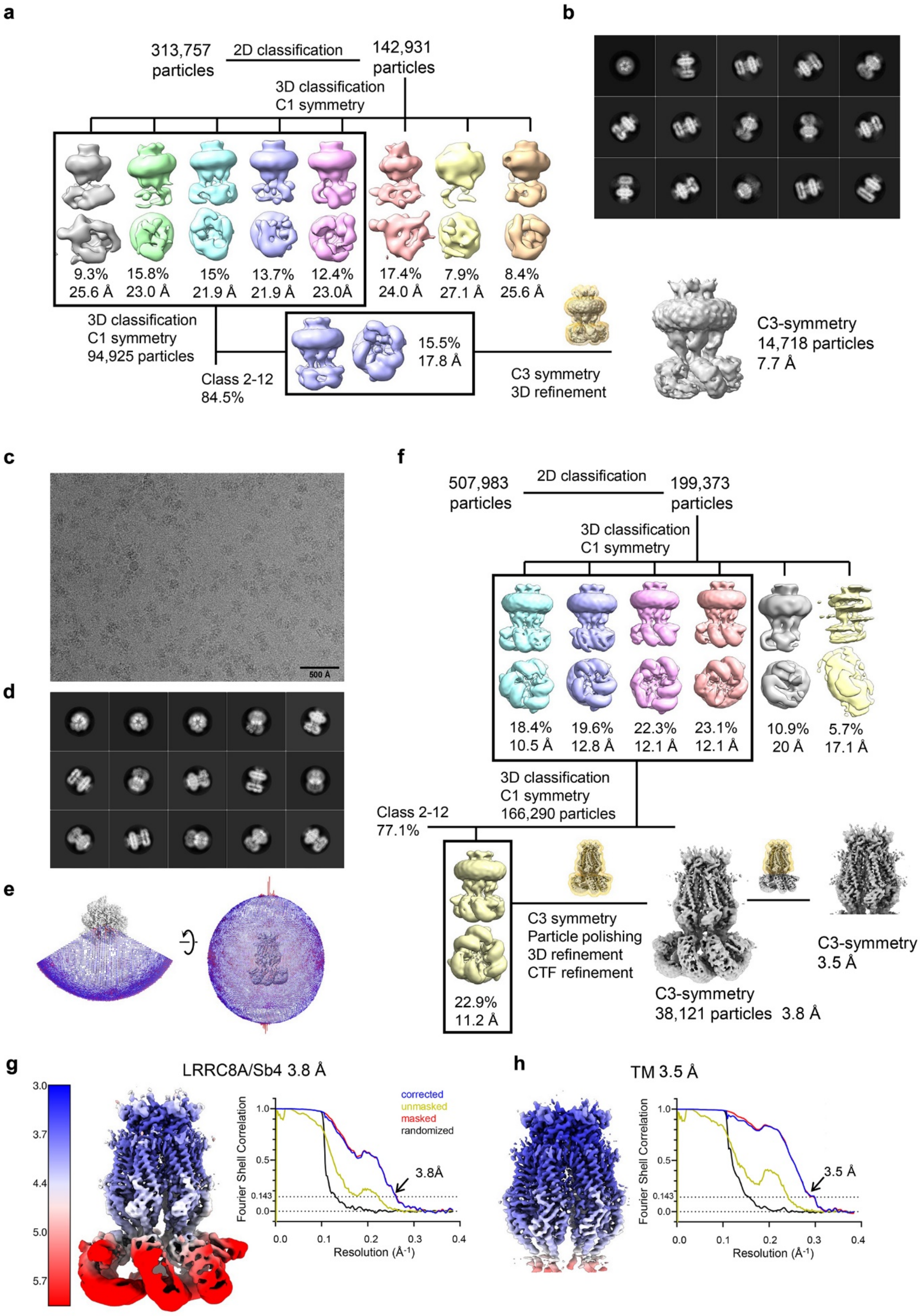
a
313,757 particles
2D classification
142,931 particles
3D classification C1 symmetry
9.3% 25.6 Å
15.8% 23.0 Å
15% 21.9 Å
13.7% 21.9 Å
12.4% 23.0Å
17.4% 24.0 Å
7.9% 27.1 Å
8.4% 25.6 Å
3D classification C1 symmetry 94,925 particles
Class 2-12 84.5%
15.5% 17.8 Å
C3 symmetry 3D refinement
C3-symmetry 14,718 particles 7.7 Å
b
c
500 Å
d
e
f
507,983 particles
2D classification
199,373 particles
3D classification C1 symmetry
18.4% 10.5 Å
19.6% 12.8 Å
22.3% 12.1 Å
23.1% 12.1 Å
10.9% 20 Å
5.7% 17.1 Å
3D classification C1 symmetry 166,290 particles
Class 2-12 77.1%
22.9% 11.2 Å
C3 symmetry Particle polishing 3D refinement CTF refinement
C3-symmetry 38,121 particles 3.8 Å
C3-symmetry 3.5 Å
g
LRRC8A/Sb4 3.8 Å
3.0
3.7
4.4
5.0
5.7
corrected
unmasked
masked
randomized
Fourier Shell Correlation
Resolution (Å-1)
3.8Å
h
TM 3.5 Å
3.5 Å

Supplementary Fig. 7: Cryo-EM structure of LRRC8A/Sb4 complexes at two different sybody ratios. **a**, **b** LRRC8A/Sb4 complex assembled at a sybody to channel subunit ratio of one. **a** Data-processing workflow. Five out of eight classes generated during 3D classification show a well-defined pore domain. The distribution of particles (%) and the resolution of each class is indicated. Particles assigned to these five classes (boxed) were pooled and used for another round of 3D classification yielding 12 classes one of which (displayed) was used for further refinement. Application of C3 symmetry resulted in a reconstruction with an overall resolution of 7.7 Å and weakly defined LRR domains with sybodies occupying alternate subunits located at r positions. **b** 2D class averages of the LRRC8A/Sb4 complex. **c–h** LRRC8A/Sb4 complex assembled at a sybody to channel subunit ratio of 0.5 (LRRC8A/$Sb4_{0.5}$). **c** Representative cryo-EM micrograph (of a total of 4,869) of the LRRC8A/$Sb4_{0.5}$ complex dataset. **d** 2D class averages of the LRRC8A/$Sb4_{0.5}$ complex. **e** Angular distribution plot of all particles included in the final C3-symmetrized reconstruction. The length and the color of cylinders correspond to the number of particles with respective Euler angles. **f** Data-processing workflow. Four out of six classes generated during the first round of 3D classification show a well-defined pore domain. The distribution of particles (%) and the resolution of each class is indicated. Particles assigned to these four classes (boxed) were pooled and used for another round of 3D classification yielding 12 classes, one of which (displayed) was used for further refinement. Application of C3 symmetry resulted in a channel with an overall resolution of 3.8 Å and weakly defined LRR domains with sybodies occupying alternate subunits located at r positions. The focused refinement of the C3 symmetric pore domain improved its resolution to 3.5 Å. Focused refinement steps of the LRR domains applying C3-symmetry or of single domain pairs with C1-symmetry did not improve the local resolution of the cryo-EM density of cytoplasmic components beyond their density observed in full-length channels and were thus omitted. Insets at different steps show the masked regions during refinement. The particle signal outside the mask was subtracted. **g–h** Final 3D reconstruction colored according to local resolution (top) and FSC plot of the final refined unmasked (yellow), masked (red), phase-randomized (black) and corrected for mask convolution effects (blue) cryo-EM density map of the LRRC8A/Sb4 complex. The resolution at which the FSC curve drops below the 0.143 threshold is indicated. **g** Full-length complex defining the location of sybodies binding to three of the six LRR domains located at the r-position of interacting domain pairs, **h** masked TM region.

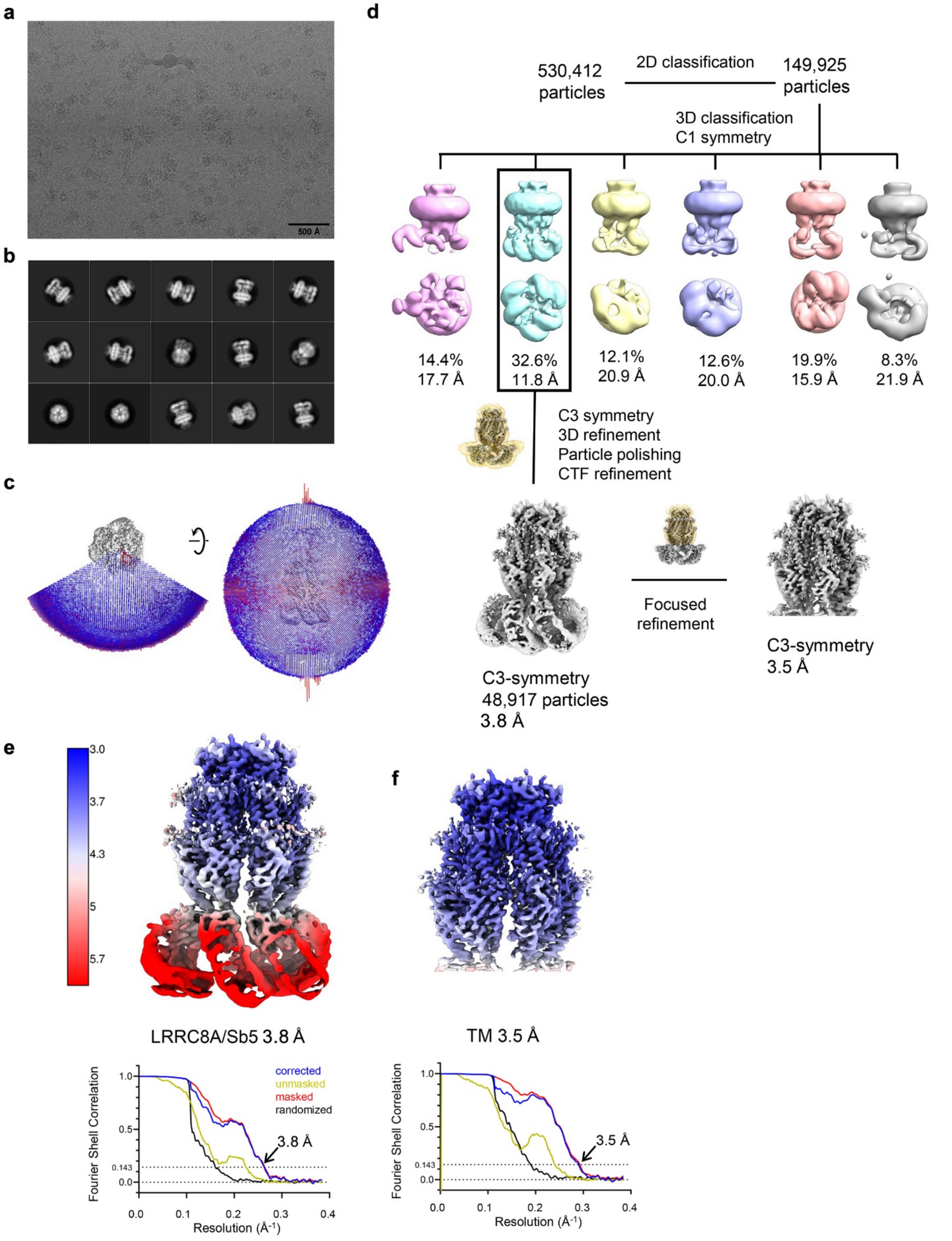

Supplementary Fig. 8: Cryo-EM structure of the LRRC8A/Sb5 complex. a Representative

cryo-EM micrograph (of a total of 5,199) of the LRRC8A/Sb5 complex dataset. **b** 2D class averages of the LRRC8A/Sb2 complex. **c** Angular distribution plot of all particles included in the final C3-symmetrized reconstruction. The length and the color of cylinders correspond to number of particles with respective Euler angles. **d** Data-processing workflow. Six classes generated during 3D classification reveal a well-defined pore domain and pronounced structural heterogeneity in the LRR domains, which reflects their intrinsic mobility. Particles assigned to the boxed class with symmetric LRR domains were used in further refinement. The distribution of particles (%) and the resolution of each class is indicated. Application of C3 symmetry and further refinement steps resulted in a channel with an overall resolution of 3.8 Å and weakly defined LRR domains. The focused refinement of the C3 symmetric pore domain improved its resolution to 3.5 Å. Focused refinement steps of the LRR domains applying C3-symmetry or of single domain pairs did not improve the local resolution of the cryo-EM density of cytoplasmic components beyond their density in full-length reconstructions and were thus omitted. Insets at different steps show the masked regions during refinement. The particle signal outside the mask was subtracted. **e–f** Final 3D reconstruction colored according to local resolution (top) and FSC plot of the final refined unmasked (yellow), masked (red), phase-randomized (black) and corrected for mask convolution effects (blue) cryo-EM density map of the LRRC8A/Sb5 complex. The resolution at which the FSC curve drops below the 0.143 threshold is indicated. **e** Full-length complex defining the location of sybodies binding to three of the six LRR domains located at the r-position of interacting domain pairs, **f** masked TM region.

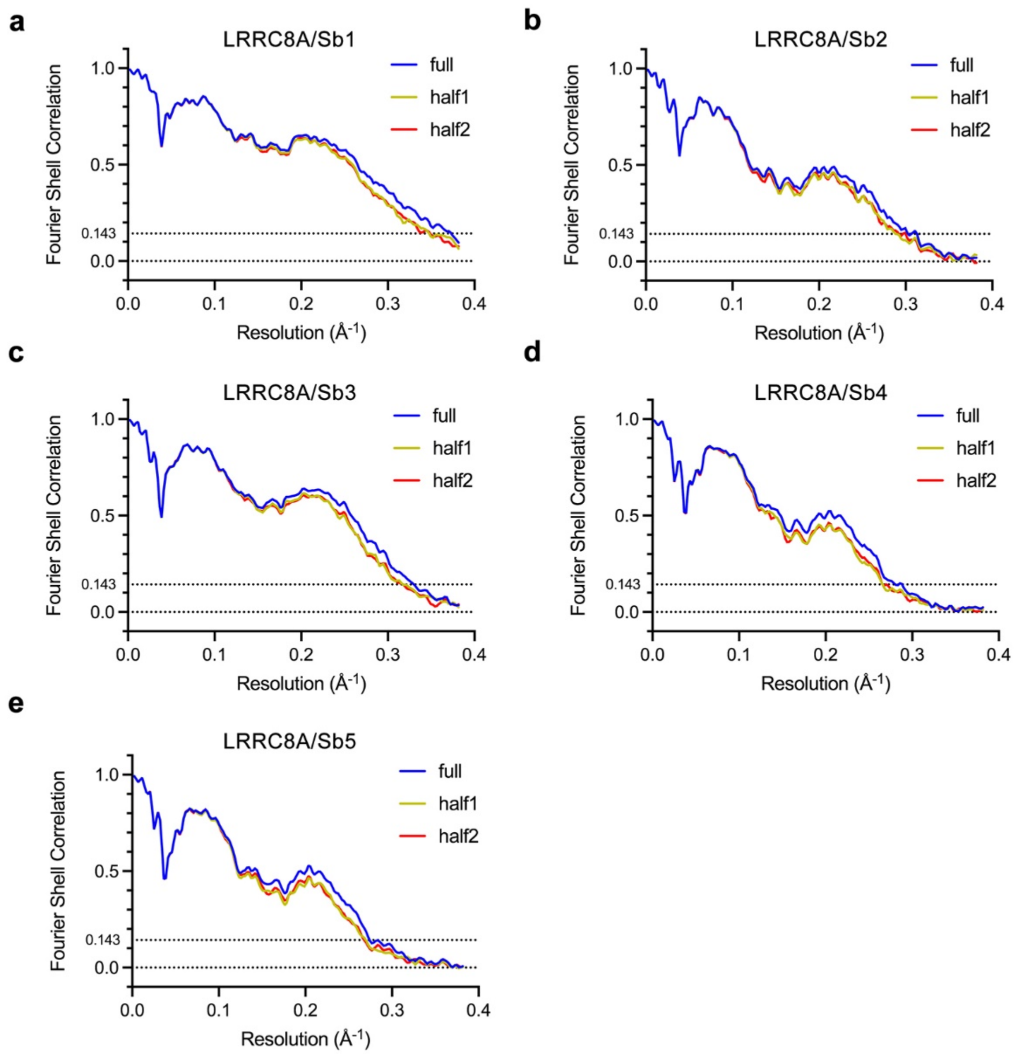

Supplementary Fig. 9: Model validation. FSC plots of refined atomic models against the cryo-EM density, full- and half-maps of full-length **a** LRRC8A/Sb1, **b** LRRC8A/Sb2, **c** LRRC8A/Sb3, **d** LRRC8A/Sb4 and **e** LRRC8A/Sb5. FSC_full (blue) was calculated for the full masked map and the model refined against the complete dataset, FSC_half1 (yellow) was calculated for the masked half-map 1 and the model refined against the dataset comprising the half map 1, FSC_half2 (red) was calculated for the masked half-map 2 and the model refined against the half map 1. The threshold of 0.143 is indicated.

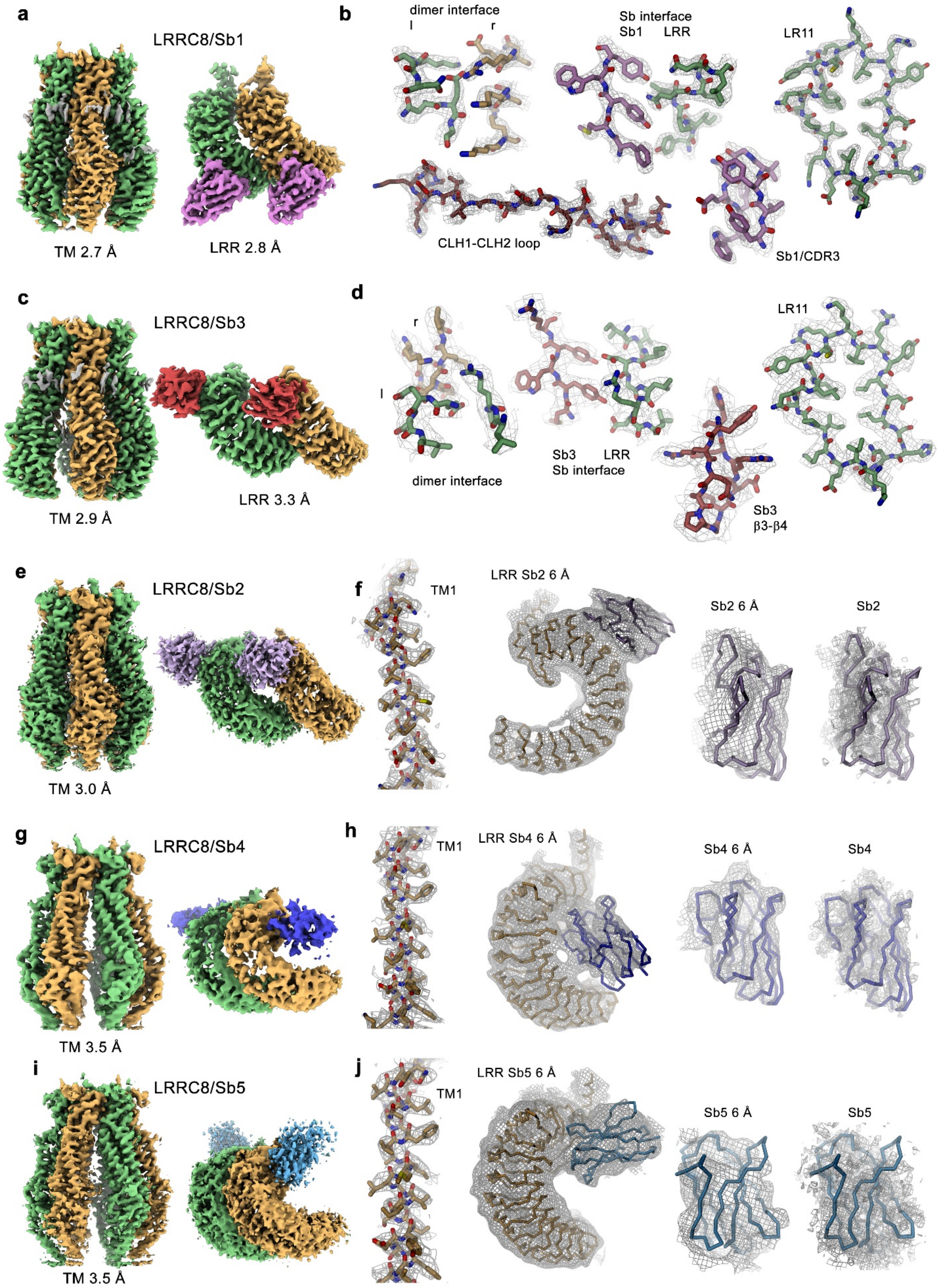
a
LRRC8/Sb1
TM 2.7 Å
LRR 2.8 Å
b
dimer interface
l
r
Sb interface
Sb1
LRR
LR11
CLH1-CLH2 loop
Sb1/CDR3
c
LRRC8/Sb3
TM 2.9 Å
LRR 3.3 Å
d
r
l
dimer interface
Sb3
LRR
Sb interface
Sb3
β3-β4
LR11
e
LRRC8/Sb2
TM 3.0 Å
f
TM1
LRR Sb2 6 Å
Sb2 6 Å
Sb2
g
LRRC8/Sb4
TM 3.5 Å
h
TM1
LRR Sb4 6 Å
Sb4 6 Å
Sb4
i
LRRC8/Sb5
TM 3.5 Å
j
TM1
LRR Sb5 6 Å
Sb5 6 Å
Sb5

Supplementary Fig. 10: Sections of cryo-EM maps. a Cryo-EM density of the LRRC8A/Sb1 complex. Left, masked refined map of the TM domain at 2.7 Å, right, masked refined map of an LRR-domain pair with bound sybodies at 2.8 Å. **b** Sections of the cryo-EM density of the masked and locally refined map of the LRR domain dimer with bound sybodies Sb1 at 2.8 Å (grey mesh) superimposed on the refined structure. The location the displayed residues is indicated. **c** Cryo-EM density of the LRRC8A/Sb3 complex. Left, masked refined map of the TM domain at 2.9 Å, right, masked refined map of an LRR-domain pair with bound sybodies at 3.3 Å. **d** Sections of the cryo-EM density of the masked and locally refined map of the LRR domain dimer with bound sybodies Sb3 at 3.3 Å (grey mesh) superimposed on the refined structure. The location the displayed residues is indicated. **e** Cryo-EM density of the LRRC8A/Sb2 complex. Left, masked refined map of the TM domain at 3.0 Å, right, LRR-domain pair extracted from the full-length map superimposed on the model. **f** Sections of the cryo-EM density of the LRRC8A/Sb2 complex. **g** Cryo-EM density of the LRRC8A/Sb4 complex. Left, masked refined map of the TM domain at 3.5 Å, right, LRR-domain pair extracted from the full-length map. **h** Sections of the cryo-EM density of the LRRC8A/Sb4 complex superimposed on the model. **i** Cryo-EM density of the LRRC8A/Sb5 complex. Left, masked refined map of the TM domain at 3.5 Å, right, LRR-domain pair extracted from the full-length map. **j** Sections of the cryo-EM density of the LRRC8A/Sb4 complex superimposed on the model. **f, h, j** Shown is the masked and locally refined map (left and right) or a map low-pass filtered to 6 Å (center). The density around TM1 (left) displays the quality of the pore domain. Low-pass filtered densities of the r subunit and its attached sybody (center left) and the isolated sybody (center right) display the envelope of the sybody, which defines its coarse location on the LRR domain. **e, g, i** The improvement of the resolution after masking of the TM domain compared to Fig. 3c–e emphasizes the quality of the data, despite the high mobility of the LRR domains. **a–j** Subunits at left and right positions in the C3 symmetric channels are colored in green and beige, respectively. Sybodies are shown in unique colors.

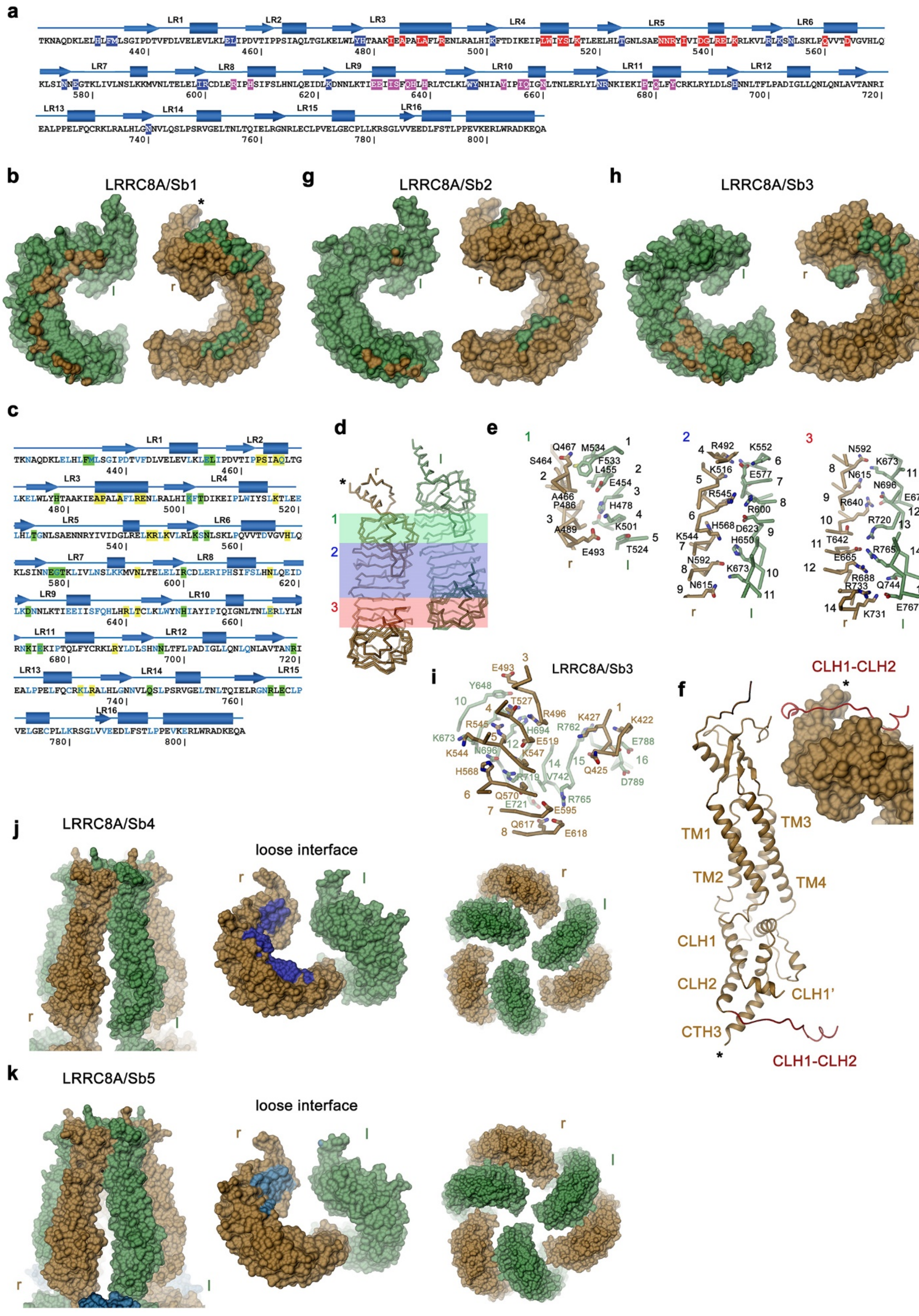
a
b
LRRC8A/Sb1
g
LRRC8A/Sb2
h
LRRC8A/Sb3
c
d
e
f
CLH1-CLH2
TM1
TM2
TM3
TM4
CLH1
CLH2
CLH1'
CTH3
i
LRRC8A/Sb3
j
LRRC8A/Sb4
loose interface
k
LRRC8A/Sb5
loose interface

Supplementary Fig. 11: Structural Features. a Sequence of the LRR domain of LRRC8A with residues involved in sybody interactions highlighted (Sb1, magenta; Sb3, red; Sb4, blue). **b** Molecular surface of two interacting LRR domains in the LRRC8A/Sb1 complex viewed as open book representation from the dimer interface. **c** Sequence of the LRR domain of LRRC8A with residues involved in the dimer interface highlighted (l-subunit, green, r-subunit, yellow). **a**, **c** Secondary structure elements and repeat numbers are indicated above, residue number below the sequence. **d** Cα trace of an interacting domain dimer viewed from the channel inside. The interface is divided into three regions that are highlighted in unique colors. **e** Sections of the domain interface as defined in **d** with protein shown as Cα trace and interacting residues labeled and shown as sticks. **f** Ribbon representation of the transmembrane domain with selected secondary structure elements labeled (left) and molecular surface of the top of the LRR domain of the r-subunit with the interacting CLH1-CLH2 loop (red) shown as ribbon (right) Asterisk marks connection between transmembrane and LRR domains. **g** Molecular surface of two interacting LRR domains in the LRRC8A/Sb2 complex and **h,** the LRRC8A/Sb3 complex viewed as open book representation from the dimer interface. **b, g, h** left and right subunits are colored in green and beige respectively, with contact areas of the interacting subunits shown in respective colors. **i** Section of the domain interface as defined in the LRRC8A/Sb3 complex with protein shown as Cα trace and interacting residues labeled and shown as sticks. **j, k** Sections of the surface of the LRRC8A channels as obtained from structures of complexes with activating sybodies. **j** LRRC8A/Sb4 complex, **k** LRRC8A/Sb5 complex. Left, view of the pore domain from the membrane shows dissociation of intracellular contacts on the intracellular part. Center, domain pair interacting at the loose interface with contact region of interacting sybodies shown in unique colors. Right, view of the LRR domains from the cytoplasm. **e**, **i** Numbers refer to the respective repeats of the LRR domain. **b, d, e, g–k** left (l) and right (r) subunits are indicated.
