# Supplementary material for: Allosteric modulation of LRRC8 channels by targeting their cytoplasmic domains
Source: Nat Commun. 2021 Sep 14;12:5435. doi: 10.1038/s41467-021-25742-w (PMC8440666; doi:10.1038/s41467-021-25742-w)
Supplement: Supplementary file 5 — Description of additional supplementary files [file 41467_2021_25742_MOESM5_ESM.docx]

Description of additional supplementary files

Title: Video 1

Description: Structural plasticity of LRRC8 channels 1. Morph between channel conformations of inhibitory LRRC8A-sybody complexes.

Title: Video 2

Description: Structural plasticity of LRRC8 channels 2. Morph between conformations of the LRRC8A-Sb1 complex and LRRC8A-Sb4,Sb5 complexes.
