# Supplementary material for: Allosteric modulation of LRRC8 channels by targeting their cytoplasmic domains
Source: Nat Commun. 2021 Sep 14;12:5435. doi: 10.1038/s41467-021-25742-w (PMC8440666; doi:10.1038/s41467-021-25742-w)
Supplement: Supplementary file 6 — Reporting Summary [file 41467_2021_25742_MOESM6_ESM.pdf]

nature portfolio

Corresponding author(s): Raimund Dutzler
Marta Sawicka

Last updated by author(s): Aug 25, 2021

Reporting Summary

Nature Portfolio wishes to improve the reproducibility of the work that we publish. This form provides structure for consistency and transparency in reporting. For further information on Nature Portfolio policies, see our Editorial Policies and the Editorial Policy Checklist.

Statistics

For all statistical analyses, confirm that the following items are present in the figure legend, table legend, main text, or Methods section.

n/a	Confirmed	
☐	☒	The exact sample size (*n*) for each experimental group/condition, given as a discrete number and unit of measurement
☐	☒	A statement on whether measurements were taken from distinct samples or whether the same sample was measured repeatedly
☐	☒	The statistical test(s) used AND whether they are one- or two-sided<br>*Only common tests should be described solely by name; describe more complex techniques in the Methods section.*
☐	☒	A description of all covariates tested
☐	☒	A description of any assumptions or corrections, such as tests of normality and adjustment for multiple comparisons
☐	☒	A full description of the statistical parameters including central tendency (e.g. means) or other basic estimates (e.g. regression coefficient) AND variation (e.g. standard deviation) or associated estimates of uncertainty (e.g. confidence intervals)
☐	☒	For null hypothesis testing, the test statistic (e.g. *F*, *t*, *r*) with confidence intervals, effect sizes, degrees of freedom and *P* value noted<br>*Give P values as exact values whenever suitable.*
☒	☐	For Bayesian analysis, information on the choice of priors and Markov chain Monte Carlo settings
☒	☐	For hierarchical and complex designs, identification of the appropriate level for tests and full reporting of outcomes
☒	☐	Estimates of effect sizes (e.g. Cohen's *d*, Pearson's *r*), indicating how they were calculated

Our web collection on statistics for biologists contains articles on many of the points above.

Software and code

Policy information about availability of computer code

Data collection	Clampex 10.6, EPU 2.7
Data analysis	Clampfit 10.7, Excel 2019, MotionCorr2 1.1.0, CTFFIND 4.1, RELION 3.0, Phenix 1.18, Coot 0.8.9.2, Chimera 1.15, DINO 0.9.6

For manuscripts utilizing custom algorithms or software that are central to the research but not yet described in published literature, software must be made available to editors and reviewers. We strongly encourage code deposition in a community repository (e.g. GitHub). See the Nature Portfolio guidelines for submitting code & software for further information.

Data

Policy information about availability of data

All manuscripts must include a data availability statement. This statement should provide the following information, where applicable:

- Accession codes, unique identifiers, or web links for publicly available datasets
- A description of any restrictions on data availability
- For clinical datasets or third party data, please ensure that the statement adheres to our policy

The three-dimensional cryo-EM density maps have been deposited in the Electron Microscopy Data Bank under accession numbers EMD-13202 (LRRC8A/Sb1), EMD-13203 (LRRC8A/Sb2), EMD-13208 (LRRC8A/Sb3), EMD-13212 (LRRC8A/Sb4), EMD-13213 (LRRC8A/Sb4_0.5) and EMD-13230 (LRRC8A/Sb5). The deposition includes maps of full-length proteins, corresponding half-maps 1 and 2, the mask used for final FSC calculation as well as relevant higher resolution maps obtained after local refinement. Coordinates for the models of full-length LRRC8A/Sb1, LRRC8A/Sb2, LRRC8A/Sb3, LRRC8A/Sb40.5 and LRRC8A/Sb5 have been deposited in the Protein Data Bank under accession numbers 7P5V, 7P5W, 7P5Y, 7P60 and 7P6K, respectively. The data from electrophysiological recordings showing the effect of sybodies on LRRC8 currents have been deposited in the Dryad database (https://doi.org/10.5061/dryad.ht76hdrgg). Source data are provided with this paper. Data can be made available upon reasonable request.

Field-specific reporting

Please select the one below that is the best fit for your research. If you are not sure, read the appropriate sections before making your selection.

☒ Life sciences ☐ Behavioural & social sciences ☐ Ecological, evolutionary & environmental sciences

For a reference copy of the document with all sections, see nature.com/documents/nr-reporting-summary-flat.pdf

Life sciences study design

All studies must disclose on these points even when the disclosure is negative.

Sample size	No sample size determination was performed. Functional experiments were performed multiple times with similar results and addition of further data did not change the conclusions of the study. Complete cryo-EM statistics are provided in Table 2, and supplementary figures 4-8.
Data exclusions	Data selection for cryo-EM studies is illustrated in Table 2 and supplementary figures 4-8. In electrophysiological experiments, leaky recordings were discarded. Otherwise no data were excluded from the analyses.
Replication	Electrophysiology data show the mean of the indicated number of biological replicates and errors are indicated. Recordings were performed multiple times from different transfections and all replications were successful.
Randomization	Randomization is not relevant for this study, as there were no groups allocated in any of the experiments.
Blinding	Not applicable as this is deemed not practically feasible.

Reporting for specific materials, systems and methods

We require information from authors about some types of materials, experimental systems and methods used in many studies. Here, indicate whether each material, system or method listed is relevant to your study. If you are not sure if a list item applies to your research, read the appropriate section before selecting a response.

Materials & experimental systems

n/a	Involved in the study
☐	☒ Antibodies
☐	☒ Eukaryotic cell lines
☒	☐ Palaeontology and archaeology
☒	☐ Animals and other organisms
☒	☐ Human research participants
☒	☐ Clinical data
☒	☐ Dual use research of concern

Methods

n/a	Involved in the study
☒	☐ ChIP-seq
☒	☐ Flow cytometry
☒	☐ MRI-based neuroimaging

Antibodies

Antibodies used	The antibodies used in this study are commercially available and include a mouse anti-LRRC8A primary antibody (Sigma, SAB1412855), mouse anti-pan-cadherin primary antibody (abcam, ab6528) and a peroxidase-conjugated goat anti-mouse secondary antibody (Jackson ImmunoResearch, 115-035-146).
Validation	The antibodies were validated by the suppliers and the validation reports are available from their websites.

Eukaryotic cell lines

Policy information about cell lines

Cell line source(s)	HEK293S GnTI– (ATCC, CLR-3022)
Authentication	No further authentication was performed for the commercially available cell line. The LRRC8-/- HEK 293 cell line was obtained from the laboratory of T.J. Jentsch. The lack of expression of LRRC8 proteins in latter was confirmed by electrophysiology and Western blots.
Mycoplasma contamination	The cell lines were tested and are free from mycoplasma contamination.
Commonly misidentified lines (See ICLAC register)	No commonly misidentified cell lines were used in this study.
